# The Mediating Role of Burnout in the Relationship Between Emotional Intelligence and Work Engagement Among Hospital Nurses: A Structural Equation Modeling Approach

**DOI:** 10.3390/nursrep15060208

**Published:** 2025-06-09

**Authors:** Bushra Alshammari, Petelyne Pangket, Awatif Alrasheeday, Nadiah Baghdadi, Sameer A. Alkubati, Dolores Cabansag, Neriza Gugoy, Sahar Mazied Alshammari, Abdulaziz Alanazi, Mohammed Dhaifallah Alanezi, Tahani Alshammari, Randy Mateo Valdez, Salman Alshammari, Laila Alharbi, Aliyu Alhaji Abubakar, Alia Alshammari, Farhan Alshammari

**Affiliations:** 1 Medical Surgical Nursing Department, College of Nursing, University of Hail, Hail 2440, Saudi Arabia; petelyne.pangket@tu.edu.sa (P.P.); s.alkubati@uoh.edu.sa (S.A.A.); d.cabansag@uoh.edu.sa (D.C.); n.gudoy@uoh.edu.sa (N.G.); r.valdez@uoh.edu.sa (R.M.V.); 2 Nursing Administration Department, College of Nursing, University of Hail, Hail 2440, Saudi Arabia; a.alrasheeday@uoh.edu.sa; 3 Nursing Management and Education Department, College of Nursing, Princess Nourah Bint Abdulrahman University, P.O. Box 84428, Riyadh 11671, Saudi Arabia; nabaghdadi@pnu.edu.sa; 4 Department of Nursing, Faculty of Medicine and Health Sciences, Hodeida University, Hodeida P.O. Box 3114, Yemen; 5 Maternal and Child Nursing Department, College of Nursing, University of Hail, Hail 2440, Saudi Arabia; 6 School of Nursing and Midwifery, Queen’s University Belfast, Medical Biology Centre, 6th Floor-Room 06.313, 97 Lisburn Rd, Belfast BT9 7BL, UK; aalanazi01@qub.ac.uk; 7 Department of Nursing, King Saud Medical City, Riyadh 11362, Saudi Arabia; 8 Medical Supplies, Purchasing and Contracts Department, Hail Health Cluster, Ministry of Health, Hail 2440, Saudi Arabia; modalanazi@moh.gov.sa; 9 Technical Support in Supply Chain Management, Hail Health Cluster, Hail 2440, Saudi Arabia; taawalshammari@moh.gov.sa; 10 Emergency Department, King Khalid Hospital, Hail 55421, Saudi Arabia; saamalshammari@moh.gov.sa; 11 Emergency Department, King Salman Specialist Hospital, Hail Health Cluster, Hail 2440, Saudi Arabia; llalharbi@moh.gov.sa; 12 Department of Management Information System, College of Businesses, University of Hail, Hail 2440, Saudi Arabia; ali.abubakar@uoh.edu.sa; 13 Department of Pharmaceutics, College of Pharmacy, University of Hail, Hail 2440, Saudi Arabia; alia.alshammari@uoh.edu.sa (A.A.); frh.alshammari@uoh.edu.sa (F.A.)

**Keywords:** burnout, emotional intelligence, work engagement, nurses, hospitals, nursing

## Abstract

**Aim:** This study aimed to explore the relationships between burnout, emotional intelligence (EI), and work engagement (WE) among hospital nurses. Specifically, it examined the mediating role of burnout in the relationship between EI and WE. **Background**: Nurses are frequently exposed to emotionally and physically demanding environments, which may lead to sustained occupational stress. Prolonged exposure to such conditions can contribute to burnout, adversely affecting both personal well-being and professional performance. EI is increasingly recognised as a protective factor that may alleviate burnout and enhance WE. **Methods**: A quantitative, cross-sectional correlational design was employed. A quota sampling technique was used to select 336 nurses working in public healthcare facilities in Ha’il, Saudi Arabia. Data were collected using standardised self-report instruments: the 14-item Shirom–Melamed Burnout Questionnaire (SMBM), the short-form Genos Emotional Intelligence Inventory (Genos EI), and the 9-item Utrecht Work Engagement Scale (UWES-9). Structural equation modelling examined associations and the mediating role of burnout between EI and WE. **Results**: EI was positively associated with WE and negatively with burnout. Burnout, in turn, was negatively associated with WE. Mediation analysis confirmed that burnout partially mediated the effect of EI on WE, indicating that EI nurses were less likely to experience burnout and more likely to remain engaged in their roles. **Discussion**: The results emphasise the role of EI in reducing burnout and enhancing WE among nurses. Burnout partially mediates this relationship, suggesting that EI influences WE both directly and indirectly. **Conclusions and Implications for Nursing**: Integrating EI training into professional development and implementing measures to reduce burnout may improve WE and retention. Policy efforts should ensure supportive work environments and adequate staffing to sustain nurse well-being.

## 1. Introduction

Nurses work under difficult conditions in hospital settings and are considered the backbone of healthcare [1]. They work long hours to attend to patients’ needs, leaving them physically [2] or mentally exhausted [3,4]. Consequently, they are prone to experiencing burnout, a psychological syndrome characterised by emotional exhaustion [3], depersonalisation [5], and a reduced sense of personal accomplishment [6]. Nurse burnout has serious consequences, including job dissatisfaction and high turnover rates, which may compromise the quality of patient care [7]. Recent reviews, such as Sipos et al. (2024) [8], underscore the global urgency of addressing burnout in the healthcare workforce and the need for targeted mitigation strategies in diverse healthcare settings.

In recent years, emotional intelligence (EI) has been recognised as crucial for success in the workplace, particularly in healthcare settings [9]. EI comprises a set of personality traits and abilities that predict emotional and social adaptation within a given environment [10]. EI is defined as the ability to recognise, understand and control one’s own emotions, as well as recognise, understand and influence the emotions of others [11]. In the context of nursing, EI refers to the ability to comprehend and respond appropriately to individual emotions during communication, tailoring words and actions to suit the emotional needs of a patient [12]. Strong EI helps nurses manage stress, make informed decisions, mitigate negative emotions and enhance their care delivery and well-being [13]. Consequently, EI is crucial in enhancing healthcare performance and in improving communication, team cohesion and patient outcomes [14].

Studies have shown that EI is strongly linked to burnout among healthcare workers [15,16]. Individuals with higher levels of EI tend to experience less emotional exhaustion, reduced depersonalisation and greater personal accomplishment compared to those with lower levels of EI [17]. Additionally, EI may indirectly mitigate burnout by reducing the frequency of workplace violence, which is another known contributor to nurse stress and burnout. However, the exact mechanism by which EI protects against burnout remains unclear [18].

Several studies have found that work engagement (WE) is related to EI [19,20,21]. WE is defined as a positive, fulfilling state characterised by vigour (energy and resilience), dedication (enthusiasm and pride), and absorption (full concentration and engrossment) [22,23]. However, limited studies have explored the relationship between WE and EI among healthcare practitioners, particularly in the context of Saudi Arabia. In Saudi Arabia, nurses working in public healthcare facilities face significant challenges, including high patient loads, long working hours, and limited resources [24,25,26,27]. These factors are often compounded by cultural expectations and a rapidly expanding healthcare system striving to meet the demands of a growing population [24]. As a result, these conditions contribute to increased stress, burnout, and reduced WE among hospital nurses [28].

This study addresses a critical research gap by analysing these relationships within the unique cultural and organisational context of Saudi Arabia, where collectivist values, hierarchical workplace structures, and gender-specific challenges may differently influence emotional regulation and engagement compared to other contexts. We draw upon the Cognitive-Motivational-Relational (CMR) theory of emotions, which provides a robust framework for understanding how cognitive appraisals and coping resources—such as EI—mediate emotional responses in high-stress environments. The CMR theory was chosen over other models due to its focus on the dynamic interplay between personal resources and environmental demands, making it particularly relevant for nurses navigating the pressures of Saudi Arabian hospitals.

By addressing this gap, this study aims to analyse the mediating role of burnout in the relationship between EI and WE among hospital nurses. Specifically, it seeks to explore how EI and burnout interact to shape nurses’ work engagement in the context of Saudi Arabia’s unique healthcare environment. The insights from this study are expected to contribute to the development of targeted interventions that enhance nurses’ well-being, reduce burnout, and ultimately improve the quality of patient care.

## 2. Literature Review and Hypothesis

### 2.1. Emotional Intelligence and WE

EI has been widely studied in organisational settings, especially in healthcare. EI is defined as the ability to perceive, assess, and manage emotions, both in oneself and in others [29]. Research suggests that EI is critical in improving interpersonal relationships, decision-making, and coping mechanisms within the workplace [14]. In the context of WE, which is defined as a positive, fulfilling state of mind characterised by vigour, dedication, and absorption [30], EI plays a pivotal role. Employees with high EI are more likely to manage their emotions effectively, enabling them to stay engaged with their work even in stressful conditions [31].

Furthermore, studies have consistently demonstrated that EI acts as a personal resource that enhances WE by fostering positive emotions, reducing emotional dissonance, and facilitating social support [14,21]. High-EI employees are better equipped to navigate complex emotional landscapes in their workplace, allowing them to maintain high levels of energy and focus. This relationship is also supported by the Job Demands-Resources (JD-R) model [32], which posits that personal resources such as EI bolster an individual’s motivational processes, thus promoting engagement.

This relationship can be explained through the CMR theory of emotions [33], which proposes that individuals with strong emotional regulation capabilities experience greater motivation and positive engagement at work [34]. Therefore, supported by both theoretical frameworks and empirical evidence, the following hypothesis is proposed:

**H1:**  *There is a positive relationship between EI and WE.*

### 2.2. Burnout and WE

Burnout is a well-recognised psychological syndrome stemming from prolonged stress in the workplace, characterised by emotional exhaustion, depersonalisation, and a reduced sense of personal accomplishment [28]. Burnout negatively impacts various job-related outcomes, including job satisfaction, commitment, and WE [35]. It leads to psychological detachment from work, diminished energy, and reduced professional efficacy, which are fundamentally at odds with the hallmarks of WE.

WE is considered the opposite of burnout, with engaged employees displaying high levels of energy, enthusiasm, and focus on their tasks. Research has consistently shown a negative correlation between burnout and WE [36], indicating that as burnout intensifies, the capacity for positive, proactive engagement diminishes. This inverse relationship has been observed across multiple healthcare contexts [37,38], reinforcing the conceptual view that burnout directly depletes the personal resources required for engagement.

Given the consistent empirical evidence and the conceptual framework that burnout undermines the vigour and dedication central to WE, it is hypothesised:

**H2:**  *There is a negative relationship between burnout and WE.*

### 2.3. EI and Burnout

EI has been identified as a crucial factor in mitigating the effects of burnout. Research suggests a connection between EI and how individuals respond to work-related stressors, with EI being negatively associated with burnout and positively associated with engagement [39]. Early burnout research primarily focused on environmental factors as predictors [40], but over time, the role of individual characteristics has gained attention. Studies using personality variables often apply the person–organisation fit model, proposing that the greater the mismatch between an employee and their job, the higher the likelihood of burnout. Maslach and Leiter [38] argued that personal traits influence how individuals experience burnout, with attributes like personality and attribution styles playing a role. In a study of Chinese teachers, Chan [41] found that individuals with higher EI were less susceptible to burnout. This is because those with higher EI are better able to interpret emotional information, allowing them to respond to stressors more effectively and guide adaptive behaviours. According to Wong and Law [42], individuals with high EI are better equipped to regulate their emotions, handle stress, and reduce the emotional exhaustion that often leads to burnout. Research suggests that healthcare workers, including nurses, who exhibit higher levels of EI experience lower levels of burnout [17]. These findings suggest that EI may serve as a buffer against the negative emotional experiences that contribute to burnout. Thus, the following hypothesis is proposed:

**H3:**  *There is a negative relationship between EI and burnout.*

### 2.4. The Mediating Role of Burnout

One of the theoretical frameworks that explains the interplay between EI, burnout, and WE is the CMR theory [33]. This theory posits that individuals strive to manage, protect, and conserve their emotional resources, especially when confronted with workplace stressors like burnout. Burnout depletes these emotional resources, leading to reduced WE. Employees experiencing burnout are less likely to remain engaged in their work tasks due to the emotional toll and stress they face. However, individuals with high EI are better equipped to manage their emotional responses, enabling them to conserve and effectively utilise their emotional resources. This helps mitigate the impact of burnout, allowing them to maintain higher levels of WE. EI helps in reducing the emotional exhaustion and stress that often contribute to burnout, thus acting as a buffer that allows employees to remain focused and motivated in their work. In this way, burnout not only directly influences WE but also mediates the relationship between EI and WE. Employees with high EI are less likely to experience burnout, which in turn fosters higher engagement. By reducing the detrimental effects of burnout, EI plays a crucial role in sustaining WE.

**H4:**  *Burnout mediates the relationship between EI and WE.*

### 2.5. Theoretical Framework

This study is anchored in multiple complementary frameworks to explain the complex relationships among EI, burnout, and WE. Central to this is the CMR theory of emotions [33], which posits that individuals continuously appraise their environment to assess the significance of events and their coping resources. These cognitive appraisals generate emotional responses and shape subsequent behaviours. In the workplace, employees with high EI—defined as the ability to perceive, understand, and manage emotions [43]—are better able to appraise stressors as manageable and engage in effective emotional regulation, thus enhancing their motivation and engagement [33].

In parallel, the JD-R model [32,44] conceptualises how job demands (e.g., workload, emotional labour) can reduce energy, leading to burnout, while job resources (e.g., support, autonomy) and personal resources (e.g., EI, self-efficacy) promote motivation and WE. This framework underscores that burnout and engagement represent two poles of employee well-being, with EI acting as a crucial personal resource. High-EI employees are more capable of managing the emotional demands of healthcare, buffering the negative impacts of high job demands and fostering a positive motivational process that leads to engagement [32].

Empirical studies support these theoretical links. For example, Mikolajczak et al. [45] and Extremera et al. [19] found that EI significantly predicts reduced emotional exhaustion and burnout in healthcare workers. Pekaar et al. [46] further demonstrated that EI enhances WE by helping individuals proactively manage emotional challenges and seek social support. These findings align with the Conservation of Resources (COR) theory [47], which posits that individuals with higher emotional resources (like EI) are better able to preserve their well-being and remain engaged under stress. Finally, Affective Events Theory [48] and the broaden-and-build theory of positive emotions [49] suggest that high-EI employees experience more positive emotions and adaptive coping strategies, further promoting engagement and mitigating burnout.

Together, these frameworks suggest that EI is not only directly linked to WE but also indirectly influences it through reduced burnout. High EI enables nurses to better manage stressful interactions and maintain the vigour, dedication, and absorption that characterise WE. By integrating these theoretical perspectives, our study examines how EI as a personal resource mediates the negative impacts of burnout and supports engagement in the demanding environment of Saudi Arabian hospitals.

## 3. Materials and Methods

### 3.1. Aim and Objectives

This study aimed to explore the relationship between burnout, EI, and WE among hospital nurses. Specifically, it examined the mediating role of burnout in the relationship between EI and WE. The study was guided by the following objectives:

1. To examine the relationship between EI and WE among hospital nurses.
2. To investigate the relationship between burnout and WE.
3. To assess the relationship between EI and burnout among hospital nurses.
4. To evaluate the mediating role of burnout in the relationship between EI and WE.

### 3.2. Design

This study used a quantitative, cross-sectional correlational design to explore the relationship between burnout syndrome, EI, and WE among hospital nurses. This design was deemed optimal because it can be leveraged to investigate the relationships between study variables effectively [50]. This manuscript adheres to the Strengthening the Reporting of Observational Studies in Epidemiology (STROBE) statement for reporting observational research.

### 3.3. Setting

Data were collected from public healthcare facilities in Hail City, Saudi Arabia, including King Salman Specialist Hospital, King Khalid Hospital, Hail General Hospital, Maternity and Children Hospital, and Shraff Hospital. These public hospitals are operated by the Saudi Ministry of Health and provide comprehensive medical treatment at no cost to Saudi nationals and non-nationals employed by the government. The sample included all nurses working in both specialised and general areas.

### 3.4. Participants/Sampling

The nurses were recruited using a quota sampling method. This method was chosen to ensure proportional representation of nurses from different hospitals and specialities within the study setting, balancing practical considerations of data collection with the need for diverse perspectives. Although quota sampling can limit representativeness compared to random sampling, it was deemed suitable for capturing variation across hospital settings and departments in this context.

After identifying the number of nurses affiliated with each health facility included in the study, G*Power software version 3.1 was utilised to calculate the appropriate sample size. With a 95% confidence level and a margin of error no greater than 5%, the software determined that approximately 278 participants were required for the study.

The study included nurses working at five target hospitals in Hail City, regardless of their wards, areas of specialisation, outpatient departments, or nursing offices. Registered nurses were eligible for inclusion in the study if they had worked for at least one year in their current departments. Participants were excluded if they were on leave, had less than one year of experience, were interns, or were nursing students. Additionally, those who declined to participate were not included in the study.

### 3.5. Questionnaire

Demographic data were collected, including age, gender, marital status, years of experience, and hospital area.

Burnout was measured using the 14-item Shirom–Melamed Burnout Questionnaire (SMBM) [51], which has demonstrated high reliability and validity across numerous studies [52]. This widely used health research tool asks respondents to indicate the frequency of recent experiences of physical fatigue, cognitive weariness and emotional exhaustion at work, with all items rated on a 7-point scale from 1 (almost never) to 7 (almost always). The total score for the SMBM is calculated by averaging the responses across all items on the 7-point scale that ranged between 7 and 98. The reliability of the SMBM was measured in the sample, yielding a Cronbach’s alpha of 0.957, indicating excellent internal consistency.

EI was measured using the short-form version of the Genos Emotional Intelligence Inventory (Genos EI) [53]. Genos EI is available as versions with 70 or 31 items; however, due to the time constraints of the participating nurses, this study selected the 14-item version. Genos EI assesses typical emotional and behavioural responses at work through seven key dimensions: self-awareness, emotional expression, emotional awareness of others, emotional reasoning, emotional self-management, emotional management of others and emotional self-control. Participants were asked to rate the frequency of their thoughts, feelings and responses using a 5-point scale, where 1 corresponds to ‘almost never’, 2 to ‘seldom’, 3 to ‘sometimes’, 4 to ‘usually’ and 5 to ‘almost always’. Scores can range from 14 to 70, with higher scores indicating higher levels of EI. The Genos EI inventory was designed for use in workplace settings with currently employed individuals, yielding scores that represent the relative frequency at which an individual engages in emotionally intelligent behaviours. The reliability of Genos EI in this study was strong, with a Cronbach’s alpha of 0.83 [54], indicating that it is a consistent and reliable tool for measuring EI in workplace contexts. The reliability of the Genos EI scale in the current sample was measured with a Cronbach’s alpha of 0.944, indicating consistent responses among participants.

WE was assessed using the Utrecht Work Engagement Scale 9-item version (UWES-9) (20) rather than the UWES-17 to accommodate the study’s use of three different scales, thereby reducing the time required for respondents to complete the questionnaires and increasing the response rate. The UWES-9 is a nine-item self-report scale divided into three subscales, each containing three items: vigour (VI), dedication (DE) and absorption (AB). All items were scored on a seven-point frequency scale ranging from 0 (never) to 6 (always). The scores ranged from 0 to 54. The UWES-9 categorises average item scores based on different engagement levels: ‘very low’ for scores of 1.77 or lower, ‘low’ for scores between 1.78 and 2.88, ‘average’ for scores between 2.89 and 4.66, ‘high’ for scores between 4.67 and 5.50 and ‘very high’ for scores of 5.51 and above. Therefore, an average score of 6 indicates very high WE. The nine-item UWES is a widely used instrument for measuring WE and has demonstrated good reliability and validity [37]. The reliability for the current sample was measured with a Cronbach’s alpha of 0.924, indicating acceptable internal consistency.

### 3.6. Data Collection

Data were collected between January and April 2024 after obtaining approval from the University of Hail Ethics Review Committee. Coordination with the hospital administration ensured staff were properly informed about the study. Consenting nurses received a Google link to the questionnaire via email, which included comprehensive study information, the researchers’ contact details in the event that any clarifications were required, a consent form and the questionnaire itself. The instructions emphasised that participants’ confidentiality and anonymity would be maintained, and they held the right to withdraw from the study at any time without consequences.

To boost participation, reminder emails were sent every two weeks. Nursing supervisors and department heads encouraged participation and facilitated access to the online survey for eligible nurses to ensure effective dissemination of the questionnaire. Researchers collaborated with hospital administrators to ensure the effective dissemination of the questionnaire to eligible nurses.

### 3.7. Data Analysis

Data analysis was performed using the Statistical Package for the Social Sciences (SPSS) version 21.0 (IBM Corporation, Armonk, NY, USA) and Analysis of Moment Structures (AMOS) 22.0 (IBM Corporation, Armonk, NY, USA). Categorical variables are summarised as frequencies and percentages, while continuous variables are presented as means and standard deviations. Associations between sociodemographic variables, participants’ characteristics and the scores corresponding to participants’ burnout, EI, and WE were determined using independent *t*-tests and ANOVA. Multiple linear regression was used to identify significant predictors of burnout and WE. Correlations between burnout, EI, and WE were assessed using Pearson’s correlation. A structural equation modelling (SEM) approach using the bootstrap method (2000 replications, 95% bias-corrected confidence intervals) was employed in AMOS 22.0 to test the mediating effect of burnout on the relationship between EI and WE. Hypotheses were tested to explore how EI potentially influences burnout and WE and to determine the indirect pathway through which EI potentially influences WE levels via burnout. With the aid of SmartPLS version 4.1.0.0, the intricate measurements and structural model analyses were carried out. Evaluation of the measurement model.

Tests for discriminant validity, convergence, and reliability were used to evaluate the constructs’ validity and reliability [55]. Several metrics, such as cross-loadings, Cronbach’s alpha, average variance extracted (AVE), and composite reliability (CR) of the components, were employed to assess convergent validity. The evaluation findings are displayed in tables and figures. Every item had a loading that ranged from 0.697 to 0.911, all of which were higher than the 0.40 minimum threshold [56,57], suggesting that for meaningful interpretation, a factor loading should be greater than 0.4.

### 3.8. Ethical Consideration

Prior to the commencement of this study, ethical approval was obtained from the University of Hail Research Ethics Committee (Approval Number: H-2024-319) in January 2024. This study was conducted in accordance with all applicable ethical standards for research involving human participants. Participants were fully informed about the purpose and significance of the study, their voluntary participation, and their right to withdraw at any time without penalty. They were also assured of the confidentiality and security of their data. Written informed consent was obtained from all participants prior to data collection. Personal identifiers were removed to maintain anonymity, and all data were securely stored on an encrypted external hard drive accessible only to the research team.

## 4. Results

A total of 336 nurses participated in the study. More than half of the participants were female (180; 53.6%), their ages ranged between 21 and 39 years (261; 77.7%), and most were married (256; 76.2%). The majority of them worked in governmental hospitals (317; 94.3%), particularly in specialised areas (214; 63.7%), and had more than 10 years of experience (189; 56.3%), as illustrated in Table 1.

Regarding the descriptive results of the measured variables, participants reported a burnout score of 44.36 ± 13.88 out of a maximum of 98, reflecting moderate levels of burnout. EI was measured with a mean score of 35.35 ± 8.98, indicating medium levels within the possible range of 14 to 70. WE was assessed with a mean score of 3.29, classified as “average” within the range of 2.89 to 4.66, suggesting moderate to good levels of engagement among the nurses (Table 2).

Multiple linear regression analysis of variables as predictors of burnout, WE, and EI scores shows that the model was significant for burnout and WE (*p* < 0.001) and EI (*p* = 0.007). It accounted for 18.2% (R^2^ = 0.182, adjusted R^2^ = 0.162), 9.5% (R^2^ = 0.095, adjusted R^2^ = 0.075), and 5.2% (R^2^ = 0.052, adjusted R^2^ = 0.035) of the variance in burnout, WE, and EI, respectively (Table 3).

Compared to the reference categories, being female (*p* < 0.001), aged 40–49 or ≥50 (*p* < 0.001 and *p* = 0.007, respectively), working in specialised hospitals (*p* < 0.001), and having greater experience were significant predictors of higher burnout scores among nurses. On the other hand, compared to reference categories, being aged ≥ 50 (*p* = 0.015) and having more experience >10 years (*p* = 0.003) were significant predictors of higher scores of WE.

This study identified significant relationships between burnout, EI, and WE among hospital nurses. A significant negative correlation was observed between EI and burnout (r = −0.374, *p* < 0.001), indicating that higher levels of burnout were associated with lower EI. Similarly, WE showed a significant negative correlation with burnout (r = −0.383, *p* < 0.001). Conversely, a significant positive correlation was found between EI and WE (r = 0.627, *p* = <0.001) (Table 4).

Table 5 (validity and reliability) outlines key psychometric properties related to three constructs: EI, burnout (which might refer to burnout or another relevant factor), and WE. The values shown for factor loadings (FA), Cronbach’s alpha (CA), composite reliability (CR), and average variance extracted (AVE) provide insights into the reliability and validity of the measures used in this study.

Starting with the factor loadings (FA), these values indicate how well each item correlates with the underlying construct it is designed to measure. Generally, factor loadings above 0.70 are acceptable. For EI, the factor loadings range from 0.716 to 0.911, with all items above the threshold, meaning they strongly measure the construct. Similarly, Burnout has loadings between 0.697 and 0.855, with just one item (Burnout 7) slightly below 0.70, but still close enough to be acceptable. The WE construct also has consistently high factor loadings, ranging from 0.737 to 0.868, ensuring that each item is a good representation of WE.

In terms of Cronbach’s alpha (CA), this statistic measures internal consistency, and values above 0.70 are generally acceptable. The values for all three constructs are exceptionally high, indicating strong reliability. EI has a CA of 0.944, Burnout has 0.957, and WE has 0.924, showing that the items within each construct consistently measure the same underlying concept.

The composite reliability (CR) further confirms these reliability findings. CR values for the three constructs exceed 0.90, which indicates very high reliability. Specifically, EI has a CR of 0.952, burnout is at 0.973, and WE has a CR of 0.946, reinforcing that the measures used are consistently reliable across items.

Lastly, the average variance extracted (AVE) measures the amount of variance captured by the construct in relation to measurement error. A value above 0.50 is desirable. EI has an AVE of 0.619, burnout scores 0.669, and WE has an AVE of 0.649. These values indicate that more than half of the variance in each construct is explained by its items, further supporting the validity of the measurement tools used.

Thus, the psychometric properties for EI, burnout, and WE indicate strong reliability and validity, with high factor loadings, strong internal consistency (CA), excellent composite reliability (CR), and adequate average variance extracted (AVE). These results suggest that the constructs measured in the study are well-suited for assessing the relationships between EI, burnout, and WE among hospital nurses.

The R^2^ values in Table 6 represent the proportion of variance in the dependent variables that is explained by the independent variables in the study. These values help assess how well the model accounts for changes in the outcomes (i.e., burnout and WE).

The R^2^ value for burnout is 0.041, meaning that the independent variables in the model explain 4.1% of the variance in burnout. This is a relatively low value, indicating that only a small portion of the factors contributing to burnout are accounted for by the variables included in the study, such as EI or other potential predictors. It suggests that other, unexamined factors may play a significant role in influencing burnout levels among nurses. Although the relationship is significant, it is clear that the model has limited predictive power for burnout in this context.

The R^2^ value for WE is 0.190, meaning that 19.0% of the variance in WE is explained by the independent variables in the model. This indicates a stronger model fit compared to burnout, as nearly one-fifth of the variability in WE is attributed to factors like EI and burnout. While this still leaves a substantial portion of the variance unexplained, it suggests that the model captures some important predictors of WE, though additional variables might further improve the explanatory power.

The R^2^ values in this study reflect that the model has a stronger ability to predict WE (19.0%) compared to burnout (4.1%). While the model provides some insights into the factors that influence WE, its predictive power for burnout is relatively weak, suggesting that future research should consider additional variables to more fully understand the determinants of both burnout and WE among hospital nurses.

The Heterotrait-Monotrait Ratio (HTMT) is a measure used to assess discriminant validity in SEM. Discriminant validity ensures that constructs that are theoretically distinct from one another are empirically distinct in the data. In this context, the HTMT ratio checks whether burnout, EI, and WE are truly different constructs. The general rule of thumb is that HTMT values should be below 0.85 to ensure adequate discriminant validity.

Burnout and EI (HTMT = 0.195): The HTMT value between burnout and EI is 0.195, which is well below the 0.85 threshold. This indicates strong discriminant validity, meaning that burnout and EI are empirically distinct constructs in this study. Despite their potential correlation, the low HTMT value shows that these two variables are measuring different aspects of the nurses’ experiences.

Burnout and WE (HTMT = 0.384): The HTMT value between burnout and WE is 0.384. This is also significantly below the 0.85 threshold, confirming that burnout and WE are separate constructs. While the two may be inversely related in theory (as higher burnout typically lowers engagement), the HTMT value shows that the measures used in the study successfully differentiate between these two constructs.

EI and WE (HTMT = 0.257): The HTMT value between EI and WE is 0.257, which is also well below the 0.85 threshold. This suggests that EI and WE are distinct variables in the study. While EI is expected to positively influence WE, the low HTMT value ensures that the two are measured as different constructs.

The HTMT values in this matrix are all well below the recommended threshold of 0.85, indicating that the constructs of burnout, EI, and WE have strong discriminant validity. This means that the items in the study measure each construct independently and that there is little overlap between them. This supports the validity of the model in capturing distinct factors affecting the nurses’ work experience (Table 7).

**Figure 1:**
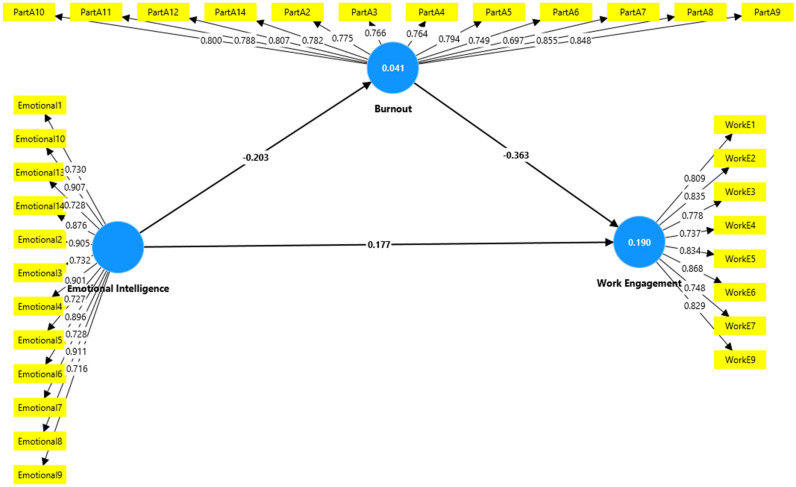
The mediating effect of EI and burnout on WE.

In the study examining the mediating effect of EI and burnout on WE among hospital nurses in Hail, Saudi Arabia, the findings can be critically discussed by considering the provided beta values, t-values, and *p*-values for both direct and mediating relationships (Figure 1).

### 4.1. The Direct Relationship

The results in Table 8 indicate significant direct relationships among the variables. Burnout showed a strong negative relationship with WE (Std. Beta = −0.363, t = 6.932, *p* < 0.001), confirming a supported relationship. EI demonstrated a significant negative relationship with burnout (Std. Beta = −0.203, t = 4.175, *p* < 0.001), which was also supported. Additionally, EI had a positive direct relationship with WE (Std. Beta = 0.177, t = 3.154, *p* < 0.001), indicating a supported positive relationship.

### 4.2. Mediating Relationship

EI → Burnout → WE: The mediating relationship shows that the effect of EI on WE was partially mediated by burnout. The beta value for the indirect path (EI → burnout → WE) was β = 0.074, with a t-value of 3.927 and a *p*-value of <0.001, providing strong support for the mediating effect (Table 9). These findings suggest that, in addition to its direct impact, EI enhances WE by mitigating the effects of burnout.

## 5. Discussion

This study investigated the relationships among EI, burnout, and WE in hospital nurses, proposing a model where burnout mediates the relationship between EI and WE. The findings confirmed the hypothesised model, demonstrating that EI has both direct and indirect effects on WE, with burnout acting as a partial mediator. These results provide critical insights into the complex interplay of these variables and their implications for healthcare professionals.

The results indicate that EI is negatively associated with burnout, while burnout negatively impacts WE. Additionally, EI was positively and significantly related to WE, both directly and indirectly. The indirect effect was mediated by burnout, confirming that burnout plays a critical role in linking EI and WE. This mediation pathway emphasises that EI not only contributes directly to higher WE but also buffers the detrimental effects of burnout, enhancing nurses’ ability to engage in their roles. These findings align with previous studies, such as the work of Pérez-Fuentes et al. [58], which highlighted that nurses with higher EI levels exhibit greater WE.

This study makes a significant contribution by validating the mediating role of burnout in the relationship between EI and WE in a Saudi Arabian hospital setting. While previous studies have confirmed the protective role of EI against burnout and its positive impact on WE, most of this research has been conducted in Western or Asian contexts [59,60]. This study extends these findings to a Middle Eastern setting, specifically within Saudi Arabia’s public healthcare system, which has distinct cultural and organisational features—such as collectivist cultural norms, hierarchical workplace structures, and gender-specific workforce challenges [61]. These cultural aspects may influence nurses’ emotional regulation and WE differently than in Western contexts. Our study, therefore, not only confirms previous findings but also contextualises them in an environment where cultural norms and healthcare delivery models shape workplace dynamics.

In Saudi Arabia, factors such as extended family support [61,62], religious and cultural expectations of gender roles [63], and a strong emphasis on community-based values have been shown to influence how individuals experience stress. These influences may be particularly relevant in the context of nurses working in Saudi hospitals, where cultural and societal expectations can amplify work-related stress. The high proportion of female nurses experiencing burnout in this study may reflect broader societal expectations and family-care obligations that add to work-related stress. Moreover, the hierarchical nature of hospital structures in Saudi Arabia may affect how nurses’ EI translates into meaningful organisational change or team-level interventions.

Similarly, Markiewicz’s [60] study demonstrated that burnout mediates the relationship between EI and stress coping strategies, further substantiating the critical role of burnout as an intermediary variable. Another study showed that trait EI mitigates the impact of negative emotions on burnout [64]. Specifically, emotions associated with anger and sadness were linked to higher levels of burnout among nurses with low trait EI, whereas this association was not observed in nurses with high trait EI. The present study reinforces these findings by providing empirical support for the partial mediation model, highlighting how burnout can amplify or diminish the benefits of EI on WE.

Burnout represents a significant burden for both organisations and individuals, leading to adverse outcomes such as job dissatisfaction, low organisational commitment, diminished job performance, and high employee turnover [65]. It also negatively impacts employees’ physical and psychological health, often manifesting as disengagement, where individuals detach from job tasks and customer interactions, shifting their attitudes from proactive care to neglect [66]. This neglect includes passive behaviours such as reduced interest in work, lateness, absenteeism, and increased error rates [67], all of which are closely tied to diminished performance.

Burnout’s effects vary depending on its dimensions. For instance, a study by Magnano et al. [67] found that disillusion—a burnout profile characterised by a lack of challenge, loss of interest in tasks, cynicism, and dissatisfaction [68]—directly influences outcomes such as turnover intentions and neglect. Disillusioned individuals are more likely to disengage or consider alternative job options, bypassing EI’s ability to buffer the negative effects of burnout.

On the other hand, EI has been recognised as a critical factor in achieving successful performance outcomes [69]. These findings are consistent with recent research conducted in the Saudi Arabian healthcare context, such as Turjuman et al. [70], which reported that EI was significantly associated with higher WE among nurses in Riyadh. Similarly, research by Nel and De Villiers [71] demonstrates that individuals with high EI tend to experience more favourable workplace outcomes. Carmeli [72] further explained that EI significantly reduces employees’ withdrawal intentions by enabling them to effectively manage and regulate their emotions. Individuals with high EI are better equipped to handle negative emotional states, minimising the likelihood of destructive consequences. Their advanced emotional regulation skills help them cope with challenges more effectively, reducing tendencies toward withdrawal or disengagement.

Contrary to earlier studies that suggested EI as a mediator between burnout and organisational outcomes [67]—such as job dissatisfaction, low organisational commitment, absenteeism, neglect, and turnover intentions—the current study identifies burnout as the mediator in the relationship between EI and WE. The findings demonstrate that nurses with high EI experience significantly lower levels of burnout across its various dimensions, which in turn enhances their WE. This suggests that emotionally intelligent individuals are better equipped to manage burnout, ultimately fostering higher engagement. This nuanced perspective highlights the pivotal role of burnout in workplace dynamics, particularly in emotionally demanding environments like hospitals, where managing emotions and mitigating burnout are essential for sustaining engagement and performance.

## 6. Implications for Nursing and Health Policy

This study presents several practical suggestions to enhance healthcare practices and delivery by increasing support for nurses. First, EI can be developed through targeted training programs, which may positively impact mental and physical health, work performance, and interpersonal relationships. Second, hospitals should implement programmes to prevent burnout and promote WE by providing supportive working conditions and recognising the efforts of their staff. Third, policymakers are encouraged to develop legislation mandating adequate staffing levels, as excessive workloads can hinder nurses’ ability to deliver high-quality care, negatively impacting patient outcomes. Finally, accessible mental health resources should be prioritised to support nurses’ psychological well-being.

Building on these practical recommendations, this study also emphasises the importance of culturally sensitive and evidence-based interventions tailored to the Saudi healthcare context. Hospital managers should prioritise EI training initiatives that account for local cultural norms, gender roles, and organisational hierarchies, recognising how these factors shape nurses’ experiences of burnout and engagement. Policymakers are urged to support and mandate EI development programs in healthcare settings and to promote policies that ensure balanced workloads, access to mental health resources, and flexible work arrangements—especially for female nurses balancing work and family roles. Initiatives that foster open communication and reduce stigma surrounding burnout can further strengthen these efforts, contributing to healthier work environments and better patient outcomes.

Theoretically, this study extends the application of established frameworks, including the JD-R and CMR models, by demonstrating the mediating role of burnout and the protective function of EI in the Saudi Arabian healthcare context. These findings underscore the need to consider cultural and organisational factors when examining workplace dynamics in non-Western settings. Practically, this study highlights the crucial role of EI as a personal resource for nurses, offering clear directions for policy and organisational strategies aimed at mitigating burnout and fostering engagement. These culturally nuanced and evidence-informed strategies are essential for improving nurse well-being, enhancing patient care, and ensuring sustainable nurse retention in Saudi hospitals.

## 7. Limitations

The cross-sectional design of this study limited its ability to establish causal relationships between burnout, EI, and WE. Longitudinal studies are required to provide clearer evidence and confirm these relationships over time. Additionally, the study’s findings may not be generalisable to other countries with different healthcare systems and cultural contexts, given that the sample was drawn from Ha’il City, Saudi Arabia. The use of self-reported questionnaires may also introduce response bias, as participants could overestimate or underestimate their levels of burnout, EI, and WE. In particular, social desirability bias may have influenced participants to respond in ways that align with professional standards or perceived expectations rather than their true experiences. This potential bias could have led to an underreporting of burnout and an over-reporting of emotional intelligence and WE, thereby affecting the study’s findings. Additionally, as this study relied solely on self-report measures, there is a possibility of common method variance (CMV) contributing to inflated relationships among these variables. Although we assured participants of confidentiality and anonymity to reduce such biases, future studies should consider using objective or multi-source data to further address CMV risk and strengthen the validity of the findings.

Another limitation of this study is the absence of specific questions regarding whether nurses had direct patient contact or held administrative roles, as well as the lack of data on the educational qualifications of the participants. Both variables could influence levels of EI, burnout, and WE, potentially affecting the study’s findings and limiting the generalisability of the results. Moreover, the study did not account for potentially important organisational factors such as organisational support, leadership styles, and work climate, which have been shown to influence burnout and work engagement. Future research should address these limitations by adopting longitudinal designs, expanding geographical scope, incorporating objective measures alongside self-reports, including a wider range of nursing professionals, and considering these additional organisational factors to enhance the reliability and relevance of the findings.

## 8. Conclusions

This study demonstrated that burnout serves as a mediating factor between EI and WE among nurses working in hospitals. Increased levels of burnout were associated with lower EI and reduced WE, indicating that higher EI might help mitigate burnout and enhance nurses’ job engagement. These findings underscore the importance of prioritising burnout prevention by enhancing EI and fostering WE among nurses. This can be achieved through targeted EI training programs, burnout prevention strategies, adequate staffing levels, and providing mental health support services tailored to the needs of nurses.

Theoretically, this study contributes to the existing literature by providing empirical evidence of the mediating role of burnout in the relationship between EI and WE. It expands the understanding of how emotional resources, such as EI, can buffer the negative effects of burnout, thereby promoting higher engagement levels in the healthcare sector. Practically, this study offers valuable insights for healthcare policymakers and administrators on the importance of developing interventions focused on EI to enhance nurse well-being and organisational performance. For future research, longitudinal studies are recommended to establish causal relationships and explore the long-term effects of EI and burnout on WE. Additionally, further research could investigate other potential mediators or moderators, such as organisational culture or social support, to provide a more comprehensive understanding of the factors influencing nurse engagement.

## Figures and Tables

**Table 1:** Socio-demographic characteristics of participants.

Characteristics		n	%
Sex			
	Male	156	46.4
	Female	180	53.6
Age			
	21–39	261	77.7
	40–49	56	16.7
	≥50	19	5.7
Marital status			
	Single	80	23.8
	Married	256	76.2
Hospital area	General Areas	214	63.7
	Specialized Areas	122	36.3
Experience in years			
	<2	42	12.5
	2–5	49	14.6
	6–10	56	16.7
	>10	189	56.3

**Table 2:** Levels of burnout, WE, and EI.

Variables	Mean ± SD
Burnout	44.36 ± 13.88
WE	29.68 ± 11.80
EI	35.35 ± 8.98

**Table 3:** Multiple regression of factors affecting nurses’ burnout, WE, and EI.

Variables		Burnout *			WE **			EI		
		β	95% CI for β	*p*-Value	β	95% CI for β	*p*-Value	β	95% CI for β	*p*-Value
Sex										
	Male	Reference			Reference					
	Female	11.990	8.94–15.04	<0.001	−0.425	−3.32–2.47	0.773			
Age										
	21–39	Reference			Reference			Reference		
	40–49	7.057	2.91–11.19	<0.001	−0.330	−4.28–3.62	0.870	1.548	−1.26–4.36	0.280
	≥50	8.512	2.35–14.66	0.007	7.258	1.40–13.10	0.015	−1.673	−6.01–2.66	0.448
Hospital area										
	General	Reference						Reference		
	Specialized	14.801	8.46–21.14	<0.001				−1.620	−6.03–2.79	0.471
Experience in years										
	<2	Reference			Reference			Reference		
	2–5	−10.464	−15.93–4.99	<0.001	−0.698	−5.83–4.44	0.790	1.982	−1.79–5.76	0.303
	6–10	−7.011	−12.31–1.70	0.010	4.341	−0.54–9.22	0.082	5.018	1.29–8.74	0.008
	>10	−9.938	−14.95–4.92	<0.001	7.192	2.50–11.88	0.003	4.525	1.22–7.82	0.007

Work Engagement = WE, * R^2^ = 0.182, Adjusted R^2^ = 0.162, *p* = <0.001; ** R^2^ = 0.095, Adjusted R^2^ = 0.075, *p* = <0.001, Emotional Intelligence = EI.

**Table 4:** Correlation matrix between the studied variables.

Variables		Burnout	EI	WE
Burnout	Pearson’s r	-	-	-
	*p*-value	-	-	-
EI	Pearson’s r	−0.374 **	-	-
	*p*-value	<0.001	-	-
WE	Pearson’s r	−0.383 **	0.627	-
	*p*-value	<0.001	<0.001	-

** Correlation is significant at the 0.01 level (2-tailed).

**Table 5:** Validity and Reliability.

Items	FA	CA	CR	AVE
Emotional1	0.730	0.944	0.952	0.619
Emotional10	0.907			
Emotional13	0.728			
Emotional14	0.876			
Emotional2	0.905			
Emotional3	0.732			
Emotional4	0.901			
Emotional5	0.727			
Emotional6	0.896			
Emotional7	0.728			
Emotional8	0.911			
Emotional9	0.716			
Burnout10	0.800	0.957	0.973	0.669
Burnout11	0.788			
Burnout12	0.807			
Burnout14	0.782			
Burnout2	0.775			
Burnout3	0.766			
Burnout4	0.764			
Burnout5	0.794			
Burnout6	0.749			
Burnout7	0.697			
Burnout8	0.855			
Burnout9	0.848			
WorkE1	0.809	0.924	0.946	0.649
WorkE2	0.835			
WorkE3	0.778			
WorkE4	0.737			
WorkE5	0.834			
WorkE6	0.868			
WorkE7	0.748			
WorkE9	0.829			

Factor Loadings (FA), Cronbach’s alpha (CA), Composite reliability (CR), Average variance extracted (AVE).

**Table 6:** R^2^ values.

Variables	R^2^
Burnout	0.041
Work Engagement	0.190

**Table 7:** Heterotrait-monotrait ratio (HTMT)-Matrix.

Heterotrait-Monotrait Ratio (HTMT)-Matrix	Burnout	EI	WE
Burnout		-	-
Emotional Intelligence	0.195	-	-
Work Engagement	0.384	0.257	-

**Table 8:** Direct Relationship.

Direct Relationship	Std. Beta	Std. Dev	t-Values	*p*-Values	Findings
Burnout → WE	−0.363	0.052	6.932	<0.001	Supported
EI → Burnout	−0.203	0.049	4.175	<0.001	Supported
EI → WE	0.177	0.056	3.154	<0.001	Supported

**Table 9:** Mediating Relationship.

Mediating Relationship	Std. Beta	Std. Dev	t-Values	*p*-Values	Findings
EI → Burnout → WE	0.074	0.019	3.927	<0.001	Supported

## Data Availability

The data presented in this study are available on request from the corresponding author. The data are not publicly available due to ethical and privacy restrictions, as they contain sensitive information pertaining to individual participants.

## Public Involvement Statement

No public involvement in any aspect of this research.

## Guidelines and Standards Statement

This manuscript was drafted against the *STROBE (Strengthening the Reporting of Observational Studies in Epidemiology)* guidelines for observational research (von Elm et al., 2007) [73]. The STROBE checklist was used to ensure comprehensive and transparent reporting of the study’s design, methods, results, and interpretation.

## Abbreviations

The following abbreviations are used in this manuscript:

EI	Emotional Intelligence
WE	Work Engagement
SMBM	Shirom–Melamed Burnout Measure
UWES	Utrecht Work Engagement Scale
CMR	Cognitive-Motivational-Relational Theory
AMOS	Analysis of Moment Structures
AVE	Average Variance Extracted
CR	Composite Reliability
CA	Cronbach’s Alpha
FA	Factor Loadings
HTMT	Heterotrait–Monotrait Ratio
